# Injectable Contraceptive Use in Women With Intellectual Disability – A Narrative Review and Local Case Series

**DOI:** 10.1192/bjo.2025.10472

**Published:** 2025-06-20

**Authors:** Marianne Bergman, Alaa Martin

**Affiliations:** 1 Essex Partnership Foundation Trust, Wickford, United Kingdom; 2 Hertfordshire Partnership Foundation Trust, Hatfield, United Kingdom

## Abstract

**Aims:** Intellectual Disability is defined as an IQ below 71. People with intellectual disability frequently experience menstrual distress leading to use of hormonal medications such as depot medroxyprogesterone acetate (DMPA). Despite risks like reduced bone mineral density (BMD) and weight gain, DMPA is widely used in this group, prompting an investigation into its suitability and associated risks.

**Methods:** A narrative review and local case series were conducted. PsycINFO and Medline were searched for articles post 1995 on contraception in people with intellectual disability post-menarche and pre menopause. The case series examined contraceptive use in 100 randomly selected menstruating people with intellectual disability. Data were collected on physical health issues. Primary care records were reviewed for contraceptive administration and risk discussions. Surveys were sent to DMPA users to assess awareness, risk understanding, and satisfaction.

**Results:** The review identified 27 papers which showed higher DMPA use in the intellectual disability population compared with the general population, and specific BMD risks. The case series found 23 people with intellectual disability using DMPA, and revealed knowledge gaps in risk and monitoring, inappropriate use given individual risk, and poor proactive risk management.

**Conclusion:** Findings indicate disproportionate DMPA use in people with intellectual disability, with inadequate clinical justification and risk awareness. Many women with intellectual disability and carers were unaware of additional BMD risks, and alternatives to DMPA were often not considered. Individualised contraceptive management and closer review of DMPA use in women with intellectual disability is needed. Monitoring could include DEXA scans, vitamin D and calcium supplementation, and weight management. Further research is needed on reasons for higher DMPA use and risks within the intellectual disability population.

